# Climate Change Influences Potential Distribution of Infected *Aedes aegypti* Co-Occurrence with Dengue Epidemics Risk Areas in Tanzania

**DOI:** 10.1371/journal.pone.0162649

**Published:** 2016-09-28

**Authors:** Clement N. Mweya, Sharadhuli I. Kimera, Grades Stanley, Gerald Misinzo, Leonard E. G. Mboera

**Affiliations:** 1 National Institute for Medical Research, Tukuyu, Tanzania; 2 Department of Veterinary Medicine and Public Health, Sokoine University of Agriculture, Morogoro, Tanzania; 3 National Institute for Medical Research, Dar es salaam, Tanzania; 4 Department of Veterinary Microbiology and Parasitology, Sokoine University of Agriculture, Morogoro, Tanzania; Institut Pasteur, FRANCE

## Abstract

**Background:**

Dengue is the second most important vector-borne disease of humans globally after malaria. Incidence of dengue infections has dramatically increased recently, potentially due to changing climate. Climate projections models predict increases in average annual temperature, precipitation and extreme events in the future. The objective of this study was to assess the effect of changing climate on distribution of dengue vectors in relation to epidemic risk areas in Tanzania.

**Methods/Findings:**

We used ecological niche models that incorporated presence-only infected *Aedes aegypti* data co-occurrence with dengue virus to estimate potential distribution of epidemic risk areas. Model input data on infected *Ae*. *aegypti* was collected during the May to June 2014 epidemic in Dar es Salaam. Bioclimatic predictors for current and future projections were also used as model inputs. Model predictions indicated that habitat suitability for infected *Ae*. *aegypti* co-occurrence with dengue virus in current scenarios is highly localized in the coastal areas, including Dar es Salaam, Pwani, Morogoro, Tanga and Zanzibar. Models indicate that areas of Kigoma, Ruvuma, Lindi, and those around Lake Victoria are also at risk. Projecting to 2020, we show that risk emerges in Mara, Arusha, Kagera and Manyara regions, but disappears in parts of Morogoro, Ruvuma and near Lake Nyasa. In 2050 climate scenario, the predicted habitat suitability of infected *Ae*. *aegypti* co-occurrence with dengue shifted towards the central and north-eastern parts with intensification in areas around all major lakes. Generally, model findings indicated that the coastal regions would remain at high risk for dengue epidemic through 2050.

**Conclusion/Significance:**

Models incorporating climate change scenarios to predict emerging risk areas for dengue epidemics in Tanzania show that the anticipated risk is immense and results help guiding public health policy decisions on surveillance and control of dengue epidemics. A collaborative approach is recommended to develop and adapt control and prevention strategies.

## Introduction

Dengue is the most important arboviral infection in the world [1,2]. The disease is endemic in more than 100 countries [3,4] and continue to spread worldwide due to emerging ecological distribution areas of disease vectors and the global movement of humans [5]. Climate has a significant role in mosquito distribution due to emerging favourable conditions especially rising temperatures [6–8]. Globally, climate change projections indicate an increase in mean temperatures ranging between 1.8°C and 4°C by the end of the 21st century [9]. In Tanzania, temperatures are expected to rise between 1°C to 3°C above baseline, and precipitation and extreme events are expected to increase in frequency by the 2050s [10]. Climate change is likely to affect human health from impacting food insecurity and malnutrition to increasing in risk areas for vector-borne diseases [11,12]. Climatic change creates new ecological niches for vectors hence altering temporal and spatial distribution of vector-borne disease [13–18]. Climate change has been implicated as a contributing factor in dengue globally [18–21] and the first outbreak of Chikungunya virus in temperate climate [8]. Studies indicate that climate change and variability influence dynamics and potential spatio-temporal distribution of dengue vectors and hence potential of disease endemicity [22–27].

In Tanzania, dengue outbreaks have been reported repeatedly in 2010, 2012 [28–30], 2013 and 2014 [31]. The most important vector in Tanzania is *Ae*. *aegypti* which is found in urban environments and prefers feeding on humans and the virus circulation is maintained through transovarial transmission [24,32]. *Ae*. *aegypti* usually bites in shady areas during the day or when the weather is cloudy, but biting is significantly high two hours after sunrise and before sunset [33]. Transmission of DENV to humans by mosquitoes involves complex processes influenced by mosquito genetics, viral genetics and bioclimatic factors [34]. The climatic conditions in which the immature mosquito larvae develop have a particularly large influence on the viral susceptibility and transmission capability of adult female mosquitoes [35–37].

Ecological Niche Models (ENMs) use environmental-climatic factors such as temperature, precipitation, elevation and derived-normalized difference vegetation index to predict climate change effects on disease vectors distribution [38,39]. There is inadequate information on linkages of current environmental conditions with dengue epidemics and the role of future climatic conditions in determination of potential epidemic risk areas in Tanzania. Hence, we use ENMs such as Maximum Entropy Species Distribution Modelling (MaxEnt) [40] for *Ae*. *aegypti* distribution to explore the geographic distribution of *Ae*. *aegypti* in relation to dengue fever epidemics. We also aim to identify bioclimatic conditions correlated with dengue fever epidemics in Tanzania.

## Materials and Methods

### Dengue fever epidemic in Tanzania

**Fig 1:**
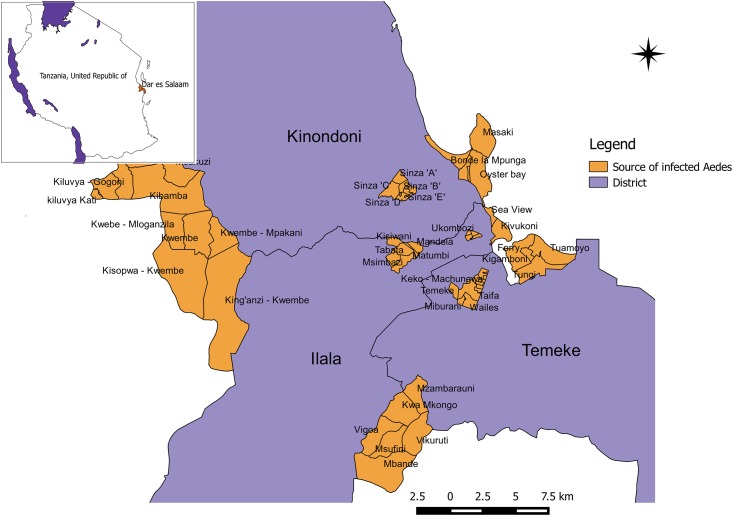
Map of Dar es Salaam indicating sites where infected *Aedes aegypti* mosquitoes with dengue virus were detected during entomological investigation following 2014 dengue epidemic.

**Fig 2:**
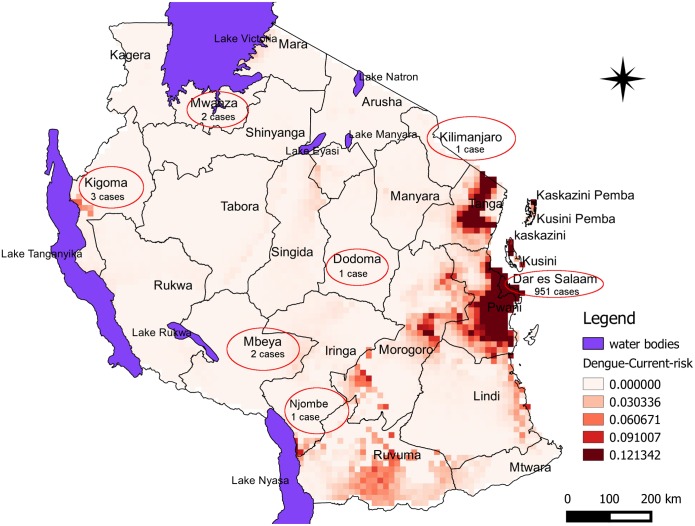
Predicted risk areas for dengue epidemics in Tanzania for the current climate scenario. The map also indicates distribution of number of dengue cases during 2014 epidemic. Colour intensification indicates increased probability of risk for dengue epidemic to occur in the area.

Following 2014 dengue epidemic in Tanzania, epidemiological and entomological investigations was carried out from May to June 2014 in Dar es Salaam [31] (Fig 1). Data on health facility-based confirmed human cases of dengue were recorded from January 2014 until end of May 2014. During that period, the disease continued spreading to different regions in the country. According to the unofficial report by the Ministry of Health and Social Welfare, a total of 961 confirmed cases were recorded in nine districts, of the seven regions in Tanzania mainland. 99% (N = 951) of the cases were from Dar es Salaam and the remaining 1% (N = 10) were reported from Mbeya (N = 2), Kigoma (N = 3), Mwanza (N = 2), Dodoma (N = 1), Kilimanjaro (N = 1) and Njombe (N = 1) regions but all with travel history to Dar es Salaam (Fig 2).

### Occurrence data for infected *Ae*. *aegypti*

*Ae*. *aegypti* mosquitoes occurrence data (S1 File**)** for both infected and uninfected with DENV was collected during a cross-sectional study conducted in Dar es Salaam from May to June 2014 [31]. During this study, adult Aedes mosquitoes were trapped using Mosquito Magnets (Mosquito Magnet Cordless Liberty Plus) [41] fixed in a total of twenty seven sentinel sites for three consecutive days in each site (Fig 1). Pre-mature stages of *Aedes* mosquitoes were collected from water containers using standard plastic dipper and then reared to adult stage. Adult mosquitoes were identified to genus or species using morphological keys [42,43]. All sampling sites were geo-referenced for the purpose of this study. Both field collected and emerged adults were killed and stored in liquid nitrogen for later screening for DENV using quantitative real time reverse transcription polymerase chain reaction (qRT-PCR).

A total of 368 mosquito pools containing ten *Ae*. *aegypti* each separated due to site of collection were ground for extraction of ribonucleic acid (RNA) using PureLink^®^ viral RNA kit (Invitrogen, Carlsbad, CA) according to manufacturer’s instructions. A qRT-PCR for the universal detection of DENV-1 to DENV-4 serotypes was performed using RealStar^®^ Dengue qRT-PCR kit version 1.0 (Altona Diagnostics GmbH, Hamburg, Germany), developed based on previous reports [44]. Of these, 330 pools were subjected to qRT-PCR for DENV detection. An overall 8.18% (N = 27) of the *Ae*. *aegypti* mosquito pools were infected with DENV [31]. Due to nature of spread of the disease, limited resources and time to conduct research to whole country, this data on infected *Ae*. *aegypti* was extrapolated to other un-sampled areas in whole country in order to identify other high risk areas under the current and predicted future climate scenarios.

### Bioclimatic variables

Environmental conditions such as temperature and rainfall influence distribution of mosquitoes and affect the ecological niche for infectious *Ae*. *aegypti* mosquitoes. Three datasets of nineteen bioclimatic variables at a spatial resolution of about 1 square kilometer were downloaded from http://www.worldclim.org/. For the current scenario, bioclimatic conditions were interpolated from observed data representative of 1950–2000. For 2020 and 2050 scenarios, downscaled global climate model (GCM) data from Coupled Model Inter-comparison Project Phase 5 (CMIP5) was used [45].

19 bioclimatic variables tested (S1 File**)** were annual mean temperature (BIO-1), mean diurnal range (BIO-2), isothermality (BIO-3), temperature seasonality (BIO-4), max temperature of warmest month (BIO-5), min temperature of coldest month (BIO-6), temperature annual range (BIO-7), mean temperature of wettest quarter (BIO-8), mean temperature of driest quarter (BIO-9), mean temperature of warmest quarter (BIO-10), mean temperature of coldest quarter (BIO-11), annual precipitation (BIO-12), precipitation of wettest month (BIO-13), precipitation of driest month (BIO-14), precipitation seasonality (BIO-15), precipitation of wettest quarter (BIO-16), precipitation of driest quarter (BIO-17), precipitation of warmest quarter (BIO-18) and precipitation of coldest quarter (BIO-19). In order to avoid the collinearity problem for MaxEnt, variables were chosen using several Jackknife procedures to identify their percentage contribution, permutation importance and relevance to infected *Ae*. *aegypti* mosquito vectors distributions as previously described [46–48]. Similar variables were also used to associate distribution of potential vectors with Rift valley fever risk areas [38,47] as well as to predict geographic distribution of triatomines infected by Triatoma virus in South America [49].

### Ecological-niche modelling

Presence record for infected *Ae*. *aegypti* co-occurrence with dengue were used together with bioclimatic data layers to develop ecological niche models. We used MaxEnt (version 3.3.1) to develop models for potential distribution of infected mosquito species in relation to reported disease cases. The dataset for all scenarios was split in the ratio of 3:1 for the training and testing respectively as previously done [40,50,51]. We used default settings for MaxEnt except that we specified a random seed with 50% of points set aside for model evaluation together with the regularization multiplier factor to reduce over-fitting due to many bioclimatic variables. Modifying the regularization multiplier helped to generate risk maps that can be extrapolated to a larger countrywide scale.

Because presence-only infected *Ae*. *aegypti* data originated from Dar es Saalam where most dengue cases were recorded, we adjusted the regularization multiplier such that predicted models results could be extrapolated to the whole country to identify other high risk un-sampled area as to the main purpose of ENMs. We used a minimum training threshold to convert raw model outputs into actual distributional estimates. The predicted distributions of risk areas are assessed by estimating the probability at maximum entropy based on assumption of uniform probability [40,52]. Twenty-seven single time occurrence records for infected *Ae*. *aegypti* tested by site of collection were used. These points were few compared to the number of total human cases records in the whole country. Despite records of few points, MaxEnt has shown to be successful in generating biologically meaningful models with occurrence records as few as six [48]. Predicted areas were identified as high risk due to probability estimates on potential occurrence of infected *Ae*. *aegypti* in the area.

### Model performance evaluation

Partial receiver operating characteristic/area under the curve (ROC/AUC) approach was used to test model predictive performance [53]. This approach does not require absence data to characterize commission errors (sensitivity). Previous models used ROC approaches that require both absence and presence data but present numerous problems [53,54]. During each prediction in this procedure, infected *Ae*. *aegypti* occurrence data was re-sampled with replacement by bootstrapping method to determine AUC ratios generated from proportion area predicted present and sensitivity. AUC ratios were calculated from observed data and random prediction. It was assumed that a good model would give AUC ratio above 1. Significance testing of model performance was done by plotting the AUC ratios replicates that would indicate a normal distribution.

## Results

### Variable importance

Of the nineteen-bioclimatic variables tested, twelve generally contributed importantly to the best model and were used as predictors for infected *Ae*. *aegypti* distributions for all climatic scenarios. Relative contributions of each variable to our prediction results by iteration of the algorithm during regularization and by random permutation in the jackknifing procedure showed that precipitation of driest month (BIO-14) and temperature annual range (BIO-7) contributed more to the model output for the current scenario whereas precipitation of warmest quarter (BIO-18) and precipitation of driest month (BIO-14) had higher permutation significance for the current scenario. For 2020 climate scenario, precipitation of driest month (BIO-14) and mean diurnal range (BIO-2) contributed more whereas precipitation of driest month (BIO-14) and precipitation of warmest quarter (BIO-18) had higher percentage on permutation importance. For 2050 climate scenario, precipitation of driest month (BIO-14) and mean diurnal range (BIO-2) contributed more whereas permutation importance was only contributed by precipitation of driest month (BIO-14), precipitation of coldest quarter (BIO-19) and precipitation seasonality (BIO-15) only. Temperature annual range (BIO-7), mean temperature of warmest quarter (BIO-10) and temperature seasonality (BIO-4) did not indicate any contribution to model output in 2050 climate scenario (Table 1).

**Table 1:** Percent contribution and permutation importance of bioclimatic variables used in the species ecological niche model.

	Current		2020		2050	
Bioclimatic variable	Contribution	Permutation	Contribution	Permutation	Contribution	Permutation
Precipitation of driest month (BIO-14)	56.6	21	61.4	50.3	59.8	53.5
Temperature annual range (BIO-7)	8.3	18.3	1.9	8.1	0	0
Mean temperature of warmest quarter (BIO-10)	5.3	0	1.1	0	0	0
Temperature seasonality (BIO-4)	5.2	3	1.4	1.5	0	0
Precipitation of warmest quarter (BIO-18)	5	53.4	6.6	21.8	9.6	0
Precipitation of coldest quarter (BIO-19)	4.4	2.4	4.7	10.8	1.1	14.9
Precipitation seasonality (BIO-15)	4.3	0.1	1.3	0	2.1	31.6
Min temperature of coldest month (BIO-6)	3.1	0	0.3	0.1	0	0
Isothermality (BIO-3)	3.0	1.7	1.7	0.6	0	0
Precipitation of driest quarter (BIO-17)	2.6	0	5.1	6.6	4	0
Max temperature of warmest month (BIO-5)	1.8	0	0.2	0.2	2	0
Mean diurnal range (BIO-2)	0.3	0	14.4	0	21.4	0

### Model performance evaluation

Model performance evaluation using commission and omission error rates based on infectious *Ae*. *aegypti* vectors occurrence points showed that it was statistically significant better than random prediction (p < 0.05) for the current and future climate scenarios. The partial ROC/AUC program generated AUC ratios ranging from 1.046 to 1.741 at the given 1- omission threshold of 0.95 using accepted omission error of 5% to the AUC at 50% for random prediction to specify the percentage of testing points which included in each of the random subsets. The models showed that the probability of the presence of infectious *Ae*. *aegypti* in relation to disease appears to be localized in the coastal areas while spreading towards the central areas of the country at high success rate according to the dengue epidemic records of 2014 (Fig 2).

### Predicted current risk areas

Model prediction results for the current scenario indicate high habitat suitability for infectious *Ae*. *aegypti* in relation to dengue epidemic to be highly localized in the coastal areas such as Dar es Salaam, Pwani, Morogoro, Tanga and Zanzibar. Risk areas also appear in Lindi and Ruvuma regions. Other areas with low probability of dengue epidemics are indicated to appear in areas around major lakes such as Lake Victoria (Mwanza and Musoma), Lake Tanganyika (Kigoma) and Lake Nyasa (Iringa and Njombe) (Fig 2). The models indicate high risk in areas previously recorded disease [28,30,55–58]. Despite records of dengue cases in Mbeya, Dodoma and Kilimanjaro, our models could not identify the area being suitable habitat for infected *Ae*. *aegypti* co-occurrence with dengue virus given that input bioclimatic variables. Prediction of low probability risk areas in Kigoma and Mwanza suggests the possibility that the disease could have occurred in the areas during the same period when epidemic was recorded in Dar es Salaam but the disease was not detected to a large scale due to lack of proper diagnosis.

### Predicted distribution of risk area at future climate scenarios

**Fig 3:**
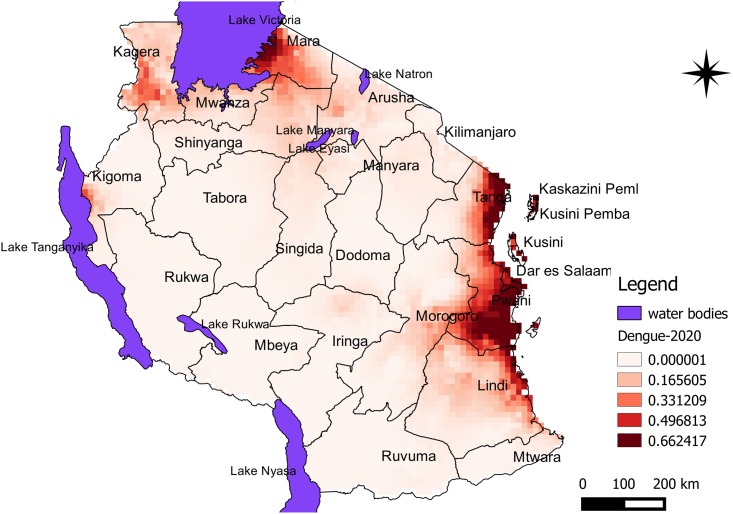
Predicted risk areas for dengue epidemics in Tanzania for the year’s 2020 climate scenario. Colour intensification indicates increased probability of risk for dengue epidemic to occur in the area.

**Fig 4:**
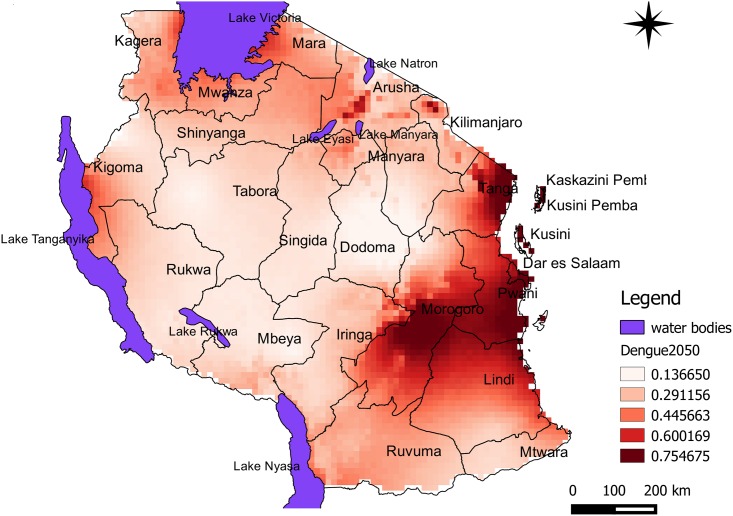
Predicted risk areas for dengue epidemics in Tanzania for the year 2050 climate scenario. Colour intensification indicate increased probability of risk for dengue epidemic to occur in the area.

Predicted risk maps for 2020 and 2050 climate scenarios show risk intensification in dengue epidemic risks areas as previously identified in the current scenario. However, for 2020, the risk seems to disappear in parts of Iringa near Lake Nyasa and Ruvuma. Dengue risk indicated broad-scale potential for change and shift in the distribution towards the central part of the country especially for 2050 projections (Figs 3 and 4). Models show anticipated high-risk areas southern parts of Lake Victoria spreading to eastern parts of Lake Tanganyika while leaving higher emerging risks in many parts of the country. Predicted suitability probability for 2050 indicated risk intensification nearly in all parts of the country (Fig 4). In general, findings indicated Dar es Salaam, Tanga, Pwani and Zanzibar would remain at high risk through 2050.

## Discussion

Despite the fact that dengue epidemics being driven by many factors including rapid urbanization and lack of adequate sanitary or mosquito control measures, climate change is a major driver of vector-borne diseases epidemics [18]. In this study, we used ecological niche models to develop risk maps of the current distribution of suitable infected *Ae*. *aegypti* habitat in Tanzania. We then use climate change predictions to determine how the change in bioclimatic factors influence potential distribution of infected *Ae*. *aegypti*, the distribution of the principal vector of dengue in relation to disease epidemic risk areas. Our results show that changing climate will expand the range of *Ae*. *aegypti* and potentially intensify risks and expand current distributions of dengue. Previous study show that climate change is happening and it is likely to expand the geographical distribution of several mosquito-borne diseases [59]. There is sufficiency evidence incriminating dengue epidemics with variations in temperature and rainfall [19,59,60]. In Tanzania, no previous studies have been conducted to predict the role of these climatic conditions towards contribution in accelerated emergence of high-risk areas for dengue epidemics.

According to the history of dengue epidemics or dengue infections in Tanzania have been reported in Dar es Salaam and Zanzibar [29,57,58]. But our models were able to indicate that during the current climate scenario, most of the eastern coastal areas were at high risk during the same period. These results suggest that dengue epidemic might have occurred in other coastal areas such as Tanga, Pwani and Morogoro but the disease was not noticed due to lack of rapid diagnostic tools and due to feverish clinical symptom of the disease that can clinically be misdiagnosed with malaria. Records of misdiagnosis of fevers associated with arboviral infections to malaria cannot be under-estimated in Tanzania [55,61].

Incorporating 2020 and 2050 climate change predictions in the niche models of infected *A*. *aegypti*, we estimate an intensification and shift of risk areas estimated from probability of occurrence of infected mosquitoes. Despite minor risk detected at the current scenario, Lake Victoria zone and northern areas of Tanzania have recorded detection of arboviruses such as Chikungunya virus and Rift Valley Fever among hospital based patients [30,62,63]. The arboviruses use nearly similar climatic drivers similar to the ones causing dengue epidemics. Trends in change for temperature, rainfall and other environmental conditions influence the length of the immature stages by providing necessary resources within the larval habitats. Model projections into 2050 conditions predict increases in intensification and distribution of high risks area nearly in all parts of the country. Future climate models scenarios show spread of disease risk from coastal areas to towards the central zones of the country while intensification in areas surrounding lake zones continues. Therefore, the identified areas should be considered when monitoring for potential future epidemics.

Despite that fewer bioclimatic variables were important predictors of the information generated by our models, their predictive performance and accuracy allows for inherited certain level of uncertainty from the modelled climate dataset that was used since the climatic conditions for the future are themselves predictions from the models [45]. Also, our ecological niche modeling approach use only one time infected mosquito occurrence records without considering other entomological parameters such as container index, Breteau index, house index and pupal index. We urge for a careful interpretation and use of results as our finding are only limited to climate change as the main driver of dengue epidemics, while other factors also present potential increase for future disease risk areas such as ecological imbalanced state as a result of habitat fragmentation, urbanization, land-use changes, and human-imposed species disequilibria, making some other areas especially susceptible to the uncertain effects of global change. These activities create favourable environmental conditions for survival of mosquito vectors hence may worsen the situation in the future. Despite these limitations, recommend surveillance for dengue fever epidemics originating in these predicted areas in the upcoming decades especially in the new suitable habitat surrounding the major lakes.

## Conclusion

Climate change represents a threat to emergence of more risk areas for dengue epidemics in Tanzania. Predicted distributions of risk areas present a cause of concern among disease managers. Results show that the influence of future climate scenarios on anticipated potential distribution of risk areas for dengue epidemic risk areas is immense and results will help in guiding future public health policy decisions on surveillance and control of dengue epidemics. A collaborative approach is recommended to develop and adapt control and prevention strategies that will help manage the anticipated risk. Concerted efforts are required towards adaptation to impact of climate change on vector-borne infectious diseases.

## Supporting Information

S1 File File containing dengue occurrence and bioclimatic data therein attached dengue-bioclimatic-data. (ZIP)
